# Different microbial genera drive methane emissions in beef cattle fed with two extreme diets

**DOI:** 10.3389/fmicb.2023.1102400

**Published:** 2023-04-13

**Authors:** Gemma A. Miller, Marc D. Auffret, Rainer Roehe, Holly Nisbet, Marina Martínez-Álvaro

**Affiliations:** ^1^Scotland’s Rural College (SRUC), Edinburgh, United Kingdom; ^2^Agrifirm, Drongen, Belgium; ^3^Institute for Animal Science and Technology, Universitat Politècnica de València, Valencia, Spain

**Keywords:** enteric methane emissions, beef cattle, concentrate-based diets, zero-grazed grass diet, microbiota by diet interaction

## Abstract

The ratio of forage to concentrate in cattle feeding has a major influence on the composition of the microbiota in the rumen and on the mass of methane produced. Using methane measurements and microbiota data from 26 cattle we aimed to investigate the relationships between microbial relative abundances and methane emissions, and identify potential biomarkers, in animals fed two extreme diets - a poor quality fresh cut grass diet (GRASS) or a high concentrate total mixed ration (TMR). Direct comparisons of the effects of such extreme diets on the composition of rumen microbiota have rarely been studied. Data were analyzed considering their multivariate and compositional nature. Diet had a relevant effect on methane yield of +10.6 g of methane/kg of dry matter intake for GRASS with respect to TMR, and on the centered log-ratio transformed abundance of 22 microbial genera. When predicting methane yield based on the abundance of 28 and 25 selected microbial genera in GRASS and TMR, respectively, we achieved cross-validation prediction accuracies of 66.5 ± 9% and 85 ± 8%. Only the abundance of *Fibrobacter* had a consistent negative association with methane yield in both diets, whereas most microbial genera were associated with methane yield in only one of the two diets. This study highlights the stark contrast in the microbiota controlling methane yield between animals fed a high concentrate diet, such as that found on intensive finishing units, and a low-quality grass forage that is often found in extensive grazing systems. This contrast must be taken into consideration when developing strategies to reduce methane emissions by manipulation of the rumen microbial composition.

## Introduction

In 2019, methane levels in the atmosphere reached record levels, about 2.5 times higher than in the pre-industrial era. Ruminants account for 16% of global methane (CH_4_) emissions, of which 35% and 30% correspond to beef and dairy industries (Tseten et al., 2022). Methane is an unnecessary by-product of microbial fermentation of mainly complex carbohydrates in the rumen, synthesized by methanogenic archaea and released into the environment through the animal’s mouth and nose. Eructated methane from ruminal microbial fermentation not only contributes to global warming, but also results in unnecessary loss of energy, compromising feed efficiency (Manzanilla-Pech et al., 2022). Because the microbiome plays a key role in the synthesis of methane, most methane mitigation strategies in ruminants rely on altering the rumen conditions and microbial ecology. Desired changes include promoting the growth of microorganisms able to reduce hydrogen (H_2_) production through propionate fermentation, re-partition H_2_ to other products by acetogenesis, inhibiting methanogens or promoting methane oxidation (Cottle et al., 2011). To this end, both short-term, e.g., use of feed-additives (Dijkstra et al., 2018; Tseten et al., 2022), and long-term strategies, e.g., microbiome-driven breeding (Martínez-Álvaro et al., 2022), have been proposed. A combination of these strategies adapted to specific production systems could be the most cost-effective solution.

In addition, implementation of strategies to reduce enteric methane emissions depends on the collection of quality methane emission data on a large-scale. Respiration chambers are the gold-standard method for measuring methane emissions from ruminants but has low throughput. Alternatives include the use of tracer gases (sulfur hexafluoride) which require boluses and attachment of equipment to the animal, and collection of multiple short-term point measurements over longer time periods (van Breukelen et al., 2022). Over the past decade, genetic sequencing costs have decreased and efficiencies have increased, resulting in rising attention to the rumen microbiome as a potential proxy measurement (Shi et al., 2014; Wallace et al., 2015; Auffret et al., 2018; Martínez-Álvaro et al., 2020). Targeted amplification of specific regions of 16S rRNA loci (16S) is a robust and cost-effective method for characterizing bacterial and archaeal community composition. Compared to whole-metagenome sequencing, it is less costly and does not require the same depth of sequencing, although it has limitations in identifying taxa at high resolution and does not provide functional information (Rubiola et al., 2022). The main statistical challenges in analyzing microbial composition data are sparsity (especially when using 16S) and their compositional nature (Gloor et al., 2017), which can lead to misleading conclusions if not addressed properly (Martínez-Álvaro et al., 2021). To circumvent these problems, appropriate techniques to exclude sparse non-informative taxa (Roesch et al., 2020) and the use of log-ratio transformations, e.g., centered log-ratio (*clr*), (Greenacre et al., 2021) have been proposed. However, these methods have only recently been utilized in microbiome studies.

Finishing diets for beef production in the UK and around the world vary in their composition, from high in concentrate (e.g., barley beef systems), to forage and grass-based diets, depending on variables such as breeding system, forage stocks, grass availability and quality, breed type, or market requirements (Agriculture and Horticulture Development Board, 2020). Both rumen microbiome composition and methane yield are highly sensitive to the diet of the animal, which determines the inputs available for microbial fermentation and the corresponding H_2_ production. Numerous studies report that the microbiome structure and methane traits are significantly altered by the forage to concentrate ratio, and by type of forage and forage quality (Janssen, 2010; Troy et al., 2015; Duthie et al., 2017; Gruninger et al., 2019; Li et al., 2019). Diet could not only cause changes in the magnitude of microbial abundances, but also alter their microbial interactions and their effects on methane emissions. Under this hypothesis, mitigation strategies or predictive equations developed for one specific diet might not be suitable for other diets.

The objectives of this study were (i) to evaluate the effects of two extreme contrasting diets (either high concentrate or low quality fresh cut grass) on methane emissions and rumen microbiota composition; and (ii) to explore the relationships between microbial abundances and methane yield and identify potential biomarkers in these two extreme feeding systems.

## Materials and methods

### Experimental design, animals, and diets

A total of 36 beef steers, 18 Limousin cross (LIMx) and 18 Aberdeen Angus cross (AAx), were selected for this trial. The cattle were paired based on breed, sire, and body weight, with one of each pair being randomly allocated to either a low quality fresh cut grass diet (GRASS) or a high concentrate total mixed ration (TMR). The 36 cattle were housed in two pens (one per treatment) and bedded on sawdust. The GRASS group had an average body weight of 499 ± 7.9 kg and the TMR group 510 ± 7.8 kg at the beginning of the trial.

All cattle had *ad libitum* access to feed and water throughout the trial. For the GRASS group, a mixed ryegrass and clover sward was mowed each morning and fed directly to the cattle top dressed with Downland Intensive Beef minerals (Downland Marketing, Carlisle, UK; 0.8% of diet dry matter). The TMR diet was mixed in a feeder wagon and had a forage to concentrate ratio of 136:864, the ingredients and nutritional composition of the diets are given in Table 1. The animals were adapted to these diets over a 2-week period, over which the proportion of concentrate or grass in the diet was gradually increased.

**TABLE 1:** Ingredients and chemical composition of two extreme offered diets fresh cut grass (Grass) and high concentrate (TMR) based.

	TMR	Grass
**Ingredient composition (g/kg DM)^1^**		
Barley	851	–
Maize dark grains	890	–
Barley Straw	865	–
Molasses	nd	–
Minerals^2^	8.1	8.0
Grass	–	210
**Chemical composition (g/kg DM)^3^**		
Dry matter (g/kg)	833	211.7
Ash	43	76
Crude protein	83.7	84.9
Neutral detergent fiber	339	557
Starch	356	<5
Metabolizable energy (MJ/kg DM)	11.7	9.0
Gross energy (MJ/kg DM)	17.9	18.1

^1^Ingredient composition is the mean of the daily diets received by the animals across the experimental period.

^2^Contained (mg/kg): Fe, 500; Mn, 3,000; Zn, 2,000; Iodine, 100; Co, 30; Cu, 800; Se, 35. Vitamins (μg/kg): vitamin A, 400,000: vitamin D3, 100,000; vitamin E, 1,500.

^3^Chemical composition is the mean of 3 analyses per treatment.

To measure methane emissions, animals were allocated to six indirect open-circuit respiration chambers over a 6-week period so that three of each treatment and three from each breed were assigned to each chamber, and paired animals were measured in the same week. Before entering the respiration chambers, animals were individually housed in training pens, which are identical in construct to the ones inside the chambers, for 6 days. After this training period, animals were isolated in the respiration chambers for 3 days, during which time they were fed once daily. Dry matter intake was recorded daily and averaged over the 3 days.

### Respiration chamber measurements

Methane measurements were undertaken in six indirect open-circuit respiration chambers and concentrations of CH_4_ in air samples exhausted from the respiration chambers were measured by infra-red absorption spectroscopy (MGA3000; Analytical Development Company Limited, Amersham, UK). The method of measurement in the respiration chambers is described in detail in Troy et al. (2015). Methane production (ppm) was determined by calculating the concentration difference between inlet and exhaust air multiplied by volumetric dry air flow and corrected to standard temperature and pressure (25°C and 101,300 Pa). Daily gas production was calculated as the average of all recorded values per chamber per day and expressed on a mass basis (g/day). The values reported in this paper are from the final 48 h of the 72-h period. Due to a technical failure of one of the respiration chambers, only 26 out of the 36 animals could be used to investigate the effects of diet type on methane yield. The remaining 26 animals were still balanced for diet treatment and breed.

### Collection of rumen samples and 16S rRNA gene analysis

There were only 26 animals with adequate rumen samples available to investigate the impact of diet on the rumen microbiome, of which 24 had methane emissions measurement, these were also still balanced for breed and diet. Rumen fluid samples were collected from each animal within 2 h of exiting the respiration chambers using a naso-gastric tube (16- by 2,700-mm Equivet stomach tube; JørgenKruuse A/S, Langeskov, Denmark) and aspirating manually. For each animal approximately 50 ml of rumen fluid was filtered through two layers of muslin and samples were stored in a −80°C freezer until sent for 16S rRNA gene analysis.

DNA was extracted from the rumen samples following the protocol of Yu and Morrison (2004) combining chemical lysis and bead beating, followed by purification on column using the QIAamp DNA Mini Kit (Qiagen, Manchester, UK). Genomic DNA was quantified using Nanodrop. Illumina TruSeq DNA libraries (Illumina Inc., San Diego, CA, USA) were prepared from genomic DNA following Illumina protocol. The V4 region of the 16S rRNA gene was amplified by PCR using Q5^®^ High-Fidelity DNA Polymerase (NEB, Hitchin, UK) and the primers 16SMiFwd and 16SMiRev, recommended by Illumina. The full sequence of the primers used were 515F = 5′-TCG TCG GCA GCG TCA GAT GTG TAT AAG AGA CAG GTG YCA GCM GCC GCG GTA A-3′ for 16SMiFwd and 806R = 5′-GTC TCG TGG GCT CGG AGA TGT GTA TAA GAG ACA GGG ACT CAN VGG GTW TCT AAT-3′ for 16SMiRev. Amplicons were purified using the ProNex^®^ Size-Selective Purification System kit (Promega, Madison, WI, USA), quantified using Qubit^®^ dsDNA HS Assay Kits (Life Technologies, Paisley, UK) prior to be pooled and sequenced on a Miseq Illumina instrument (Illumina Inc., San Diego, CA, USA) by Edinburgh Genomics (Edinburgh, UK). Sequencing provided a yield of average of 153.5 ± 47.4 Mb per sample and 5.06 ± 1.6 × 10^5^ reads per sample. The 16S rRNA amplicon sequences obtained were analyzed with the pipeline MGRAST to generate comprehensive taxonomic profiles using the standard reference database, excluding host reference genome. One table of hit counts was created for each taxonomic level, resulting in 203 genera, 115 families and 21 phyla identified.

The analysis of the taxonomic composition of the rumen microbiome was focused on (i) the natural log ratio between archaea and bacteria (A:B) and (ii) microbial composition at the genus level. The abundance of each microbial genus was normalized by the total sum of counts per sample. The microbial composition at the genus level contained a large proportion of 0 counts per sample, with an average percentage of 0 counts per sample of 78 ± 8.7% (Supplementary Figure 1). To maximize microbial information per sample, we discarded microbial genera with a high count of 0 across all samples only if they did not contribute to the discrimination of samples within diets. To achieve this, we used the Prevalence Interval for Microbiome Evaluation (PIME) workflow from the R package PIME (Roesch et al., 2020) to establish the minimum % of samples that a microbial genera should contain in order to be kept, i.e., minimum prevalence. Briefly, PIME uses a machine learning algorithm to find which is the optimal prevalence threshold to maximize the discrimination ability between GRASS/TMR diets. In our analysis, a minimum prevalence threshold of 20% maximized discriminatory ability between diets. After removing microbial genera present in less than 20% of samples, we retained 103 microbial genera for further analysis. The remaining 0 genera were imputed using a Bayesian multivariate composition approach implemented in the R package *zcompositions* (Palarea-Albaladejo and Martín-Fernández, 2015). The descriptive analysis of the rumen microbiome composition was presented in relative abundances to help their interpretability (Supplementary Figure 2). For remaining statistical analysis, the compositional nature of microbiome data was taken into account (Gloor et al., 2017) by applying a centered log-ratio (*clr*) transformation to the genera abundances, as described in Greenacre (2019) using the *zcompositions* R package.

### Data analysis

#### Effect of diet on dry matter intake, methane intensity, methane yield, and archaea: bacteria ratio (A:B)

To investigate the diet effect on DMI (kg/day), methane production (g/day), methane yield (g/kg DMI) and A:B, we fitted three different linear models including these traits as dependent variables; diet, breed, chamber, and week as fixed effects and the body weight of the animal when entering the chamber (ranging from 438 to 656 kg) as a covariate. Bayesian inference was used with bounded flat priors for all unknowns. Analyses were run with the R function *runRabbit* developed by the Institute for Animal Science and Technology from Universitat Politècnica de València.^1^ After some exploratory analyses, results were based on marginal posterior distributions of 60,000 iterations, with a burning period of 10,000 and only 1 of every 10 samples were saved for inferences. In all analyses, convergence was tested using the Z criterion of Geweke and Monte Carlo sampling errors were computed using time-series procedures (Sorensen and Gianola, 2002). The parameters obtained from the marginal posterior distributions of the differences between diets were: the mean; the highest posterior density region at 95% probability (HPD_95%_); the probability of the difference being greater than 0 when the mean is positive or lower than 0 when the mean is negative (P_0_); and the probability of the difference being greater than *r* or lower than -*r* if the mean is positive or negative (P_r_), being *r* a relevant amount having economical or biological significance. In our case, we took as *r* one-third of the phenotypic standard deviation (Blasco, 2017).

The linear relationship between methane production or methane yield and A:B within each diet was also computed. After some exploratory analysis, methane traits were analyzed within diet separately, with a model including the fixed effect of breed and the covariates body weight and A:B. In this case, we computed the mean, HPD_95%_, and P_0_ of the marginal posterior distribution of the regression coefficient between the *clr*-transformed abundance of the microbial genera and methane yield.

#### Effect of diet on the ruminal microbiome composition

A multivariate approach was used to identify which microbial genera showed different abundances due of different diets offered, using 26 samples (13 per diet group). A discriminant analysis based on projection to latent structures (DA-PLS) computed using R package *mixOmics* was fitted, considering GRASS/TMR diet treatments as dependent variables, and the 103 *clr* transformed microbial genera abundances as explanatory variables. The number of components in the model was selected based on a cross-validation procedure with 4 cross validation groups repeated 20 times, following the procedure described in Lê Cao and Welham (2022). First, we computed the Residual Sum of Squares (RSS) in the complete dataset for a given dimension *h*, using all the samples (*n* = 26):


$$R\,S\,S_{h}=\sum_{i=1}^{n}{\left(y_{i}^{\left(h\right)}-\hat{y_{i}}\right)}^{2}$$

where $y_{i}^{\left(h\right)}$ is the predicted value of sample *i* according to dimension *h* and $\hat{y_{i}}$ is its “reconstructed” value, i.e., the product between the latent component and the vector of regression coefficients for a given dimension *h.* Under each repetition, we computed the Predicted Sum of Squares (PRESS) as the sum of squares of the residuals of the testing set in each cross-validation fold:


$$PRESS_{h}=\sum_{i\;\in \;test}{\left(y_{i}^{\left(h\right)}-{\hat{y}}_{i}\right)}^{2}$$

And averaged *PRESS*_*h*_ across repetitions. The Q^2^ criterion was computed to assess the gain in prediction accuracy when one more dimension was added to the model. For each dimension:


$$Q_{h}^{2}=1-\frac{P\,R\,E\,S\,S_{h}}{R\,S\,S_{h-1}}$$

We stop adding dimensions when $Q_{h}^{2}\leq$ 0.0975 as proposed by Lê Cao and Welham (2022). The most influential variables discriminating between the two diets were selected based on their variable importance for projection (VIP) criterion >0.8 and based on their Jack Knife interval of regression coefficients not containing zero, as described in Martínez-Álvaro et al. (2021). An iterative process was followed and stopped when the removal of one more microbial genus did not improve the discrimination ability of the model. Authors are aware of the high propensity of DA-PLS models in overfitting (Westerhuis et al., 2008). We attempted to overcome this statistical issue by validating the discrimination ability of the final DA-PLS model with two different strategies. First, the final DA-PLS model was tested under a new fourfold cross validation repeated 20 times. In each fold, a DA-PLS model was re-fitted for the given set of selected variables and number of components with 3/4 of the data (training) and then used to predict to the diet of the samples in the remaining testing set (1/4 of the data). The final misclassification rate (%) was obtained by averaging the misclassification rate (%) obtained across the fourfolds in each of the 20 replicates. Second, we integrated a permutation test (randomization of diet labels in the testing set) in the currently described validation procedure as recommended by Westerhuis et al. (2008) and computed the misclassification rate (%) after permutation. It has to be realized that the same individuals in the validation set were also used to optimize the final PLS-DA model parameters (e.g., number of selected components and selected variables), and thus they are not completely independent as is requested for a proper cross validation. However, the short sample size of our study did not allowed to properly performed an external validation.

Additionally, we aimed to quantify the magnitude of the divergences between diets on the abundance of the DA-PLS selected microbial genera. To this aim, we fitted one univariate linear model for each selected microbial genera, including the fixed effects of diet and breed and body weight as a covariate. Analyses were run with the R function *runRabbit* as already described. As previously, we computed the mean, HPD_95%_, P_0_ and P_r_ the marginal posterior distributions of the differences between diets. To help interpretability, their magnitude of the differences was expressed not only as units of *clr*-transformed abundances, but also as effect size, defined as the median of the ratio of the between the difference and the variance of the traits after correction for breed, diet and body weight.

#### Associations between rumen microbiota composition and methane yield among different diets

To investigate the relationship between rumen microbiota composition and methane yield in different diets, we fitted two (one per diet group) linear projections to latent structures regression (PLS) analysis to determine the abundances of microbial genera that best explained methane yield (g/kg DMI) within each diet (*n* = 12 animals per group). Methane yield was considered as the dependent variable and the 103 *clr*-transformed abundances of microbial genera as explanatory variables. The number of components and the selection of explanatory variables in the model were chosen as explained at PLS-DA. After the final PLS model was built, its predictive ability was tested by threefold cross-validation, which was repeated 20 times. The final predictive ability of the model was calculated as the square of the correlation between the predicted and observed values in the training set, averaged over the threefolds and then over the replicates. As before, we integrated a permutation test (randomization of diet labels in the testing set) in the validation procedure and computed the predictive ability after permutation.

In addition, we aimed to study the linear regression between each PLS-selected *clr*-transformed microbial genera in each diet group and methane yield in a univariate context, to show whether a linear relationship existed between specific microbial genera and the environmental trait. To this aim, we used the data divided within diet group to fit one univariate linear model for each selected microbial genera, including methane yield as dependent variable and the fixed effect of breed and covariates of *clr*-transformed abundance of the microbial genera and body weight as covariates. Analyses were run with the R function *runRabbit* as already described. We computed the mean, HPD_95%_, and P_0_ of the marginal posterior distribution of the regression coefficient between the *clr*-transformed abundance of the microbial genera and methane yield. We additionally computed the Pearson correlation between each selected microbial genera and methane yield after correcting the data by breed and body weight.

## Results

### Effect of the diet on dry matter intake, methane traits, and A:B traits

Table 2 shows the descriptive statistics and the differences in DMI, methane traits, and A:B ratio among the diets. On average, individual animal DMI varied by ± 0.4 kg between days for GRASS animals (ranging from 0.1 to 1.3), and ± 0.7 kg for TMR animals (ranging from 0.0 to 1.5 kg). Repeatability of methane measurements between the second and third day was high, with ± 1.9 ppm average variation between days (ranging from 0.1 to 6.4 ppm). Daily dry matter intake was higher in the TMR group than in the GRASS group by 3.25 [1.04, 5.19] kg/day (*P*_0_ = 1.00). However, the TMR group had lower methane yield (−10.6 [−18.2, −1.27] g CH_4_/kg DMI) and also lower A:B (−4.30 [−6.17, −2.49]) than GRASS with *P*_0_ ≥ 0.99, and, in all cases, differences were relevant (*P*_r_ ≥ 0.98). Diet groups did not show relevant different in methane production (g/day). Breed effect did not present strong evidence of being different from zero (*P*_0_ ≤ 0.88) or relevant (*P*_r_ ≤ 0.72) for any trait.

**TABLE 2:** Means and differences between diets [fresh cut grass (GRASS) and high concentrate (TMR)] in daily dry matter intake (kg/day), methane traits and natural log archaea to bacteria ratio.

	^1^Mean TMR	^1^Mean GRASS	^2^TMR- GRASS	^3^P_0_	^4^ *r*	^5^P_r_
Daily dry matter intake (kg/day)	9.16 [8.18, 10.2]	5.95 [4.69, 7.48]	3.25 [1.04, 5.19]	1.00	0.49	0.99
Methane production (g/day)	160 [141, 181]	146 [128, 165]	14.5 [17.8, 53.4]	0.80	4.9	0.71
Methane yield (g/kg DMI)	14.4 [9.70, 19.5]	25.0 [20.7, 28.9]	−10.6 [−18.2, −1.27]	0.99	0.98	0.98
Ln (Archaea: Bacteria)	−4.72 [−5.69, −3.75]	−0.43 [−1.59, 0.58]	−4.30 [−6.17, −2.49]	1.00	0.3	1.00

^1^Means and highest posterior density intervals at 95% probability of the marginal posterior distributions of the means of TMR and GRASS.

^2^Median of the marginal posterior distribution of the difference between TMR and GRASS andighest posterior density interval at 95% probability.

^3^Probability of the marginal posterior distribution of the differences of being greater than 0 when the mean is positive or lower than 0 when the mean is negative.

^4^Relevant value considered as the minimum value with economic importance.

^5^Probability of the marginal posterior distribution of the differences of being greater than *r* when the mean is positive or lower than *r* when the mean is negative.

### Effect of diet type on the composition of the rumen microbiota at the genera level

**FIGURE 1:**
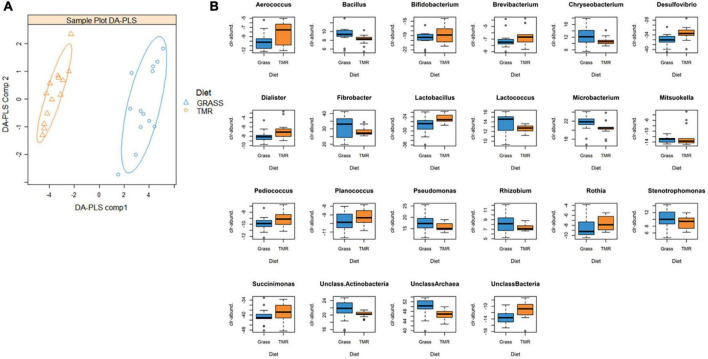
**(A)** Sample plot from a Discriminant by projection of latent structures analysis (DA-PLS) fitted to discriminate amongst the fresh cut grass (Grass) and high concentrate (TMR) fed animals based on 28 clr-transformed microbial genera abundances. The model was built with a single component and presented a miss-classification rate of 0%. For visualization purposes, sample plot is based on two components. **(B)** Microbial genera identified by DA-PLS which presented differential abundances between grass and TMR (probability of the difference of being different from 0 ≥ 0.95). Differences are expressed in units of clr-transformed abundances. Full details of the PLS analysis and linear models can be found in Supplementary Table 1.

To further examine the effects of the two extreme diets on the taxonomic composition of the rumen microbiota, we used a multivariate DA-PLS to determine which microbial genera abundances were discriminatory between the two diet groups (Supplementary Table 1; Figure 1A). We identified 28 microbial genera that, combined in a single DA-PLS component, were able to discriminate between the GRASS and TMR diets with a misclassification rate of 0% after fourfold cross-validation and 20 replicates. When the model was used to predict a randomized vector of the diet labels, it the misclassification rate increased up to 46%. Once we identified the 28 microbial genera with discrimination ability between the diets, we fitted a linear model for each microbial genus to quantify differences between diets in the *clr*-transformed abundances. Of the 28 microbial genera tested, 22 microbial genera had different abundances between the two diets (*P*_0_ ≥ 0.95, Supplementary Table 1; Figure 1B) and the differences were relevant, i.e., larger than 1/3 their standard deviation (*P*_r_ ≥ 0.90). Whilst we do not have enough power to detect differences between diets in the most ubiquitous methanogenic genera *Methanobrevibacter* and *Methanobacterium* (i.e., the HPD_95%_ were very wide [−1.93, 2.32] and [−3.19, 5.74], both including the relevant values 0.48 and 1.00), unclassified *Archaea* were much more abundant in the GRASS animals, with difference being 9.42 [6.27, 12.7] (relevant value was 0.74 and *P*_r_ = 1.00), having an effect size of 4.25. The major fiber degraders *Fibrobacter* and *Bacillus* were also more abundant in the GRASS group, as were unclassified *Actinobacteria, Microbacterium*, *Chryseobacterium*, *Lactococcus*, and three microbial genera from the Proteobacteria phylum (*Pseudomonas, Stenotrophomonas*, and *Rhizobium*), with effect sizes ranging from 1.44 to 2.63 (*P*_r_ ≥ 0.93). In contrast, *Desulfovibrio, Lactobacillus*, and unclassified Bacteria were much more abundant in the TMR group with effect sizes of −5.31, −3.64, and −3.93 (*P*_r_ = 1.00). *Bifidobacterium, Succinimonas, Aerococcus*, and *Dialister*, and *Rothia* amongst others, were also relevantly more abundant in TMR (*P*_r_ ≥ 0.93), with effect sizes ranging from −1.40 to −2.78 (see Supplementary Table 1).

### Multivariate prediction of methane yield based on abundance of microbial genera and investigation of linear microbiota-methane relationships in different diets

**FIGURE 2:**
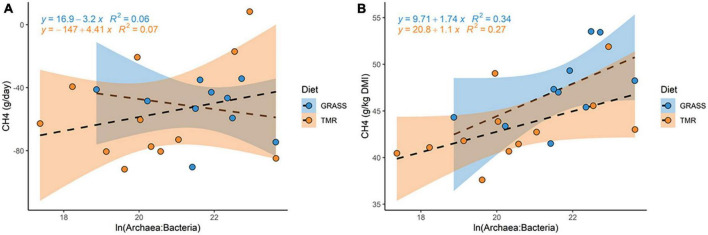
Data distribution of methane emissions [expressed as **(A)** methane production (g/day) or **(B)** methane yield (g/kg DMI)] and the natural log-ratio between archaea and bacteria abundances within animals offered two contrasting diets, high concentrate (TMR) or fresh cut grass (Grass). Methane and natural log-ratio between archaea and bacteria abundances was pre-corrected by fixed effects of breed, and body weight as a covariate.

A strong positive linear relationship between methane yield and A:B was observed in the GRASS animals, with a positive regression coefficient of +3.91 [0.28, 7.38] g CH_4_/kg DMI gain per unit A:B (*P*_0_ = 0.98). This linear relationship was also positive in animals fed TMR, although the regression coefficient was lower (0.93 [−0.45, 2.43], *P*_0_ = 0.91). When methane emission is expressed in g/day, we did not had enough evidence to stablish the sign of relationship with A:B (*P*_0_ = 0.69 in GRASS and 0.83 in TMR). Figure 2 shows the distribution of methane production (A) and methane yield (B) with A:B, after correcting the data for breed and body weight. As expected, the greatest R^2^ was obtained for methane yield and A:B in GRASS (*R*^2^ = 0.34) and TMR (*R*^2^ = 0.27), with Pearson correlations of 0.58 ± 0.21 and 0.52 ± 0.23. Based on this result, we concentrated on relationships between methane yield and rumen microbiota composition at lower taxonomic levels.

**FIGURE 3:**
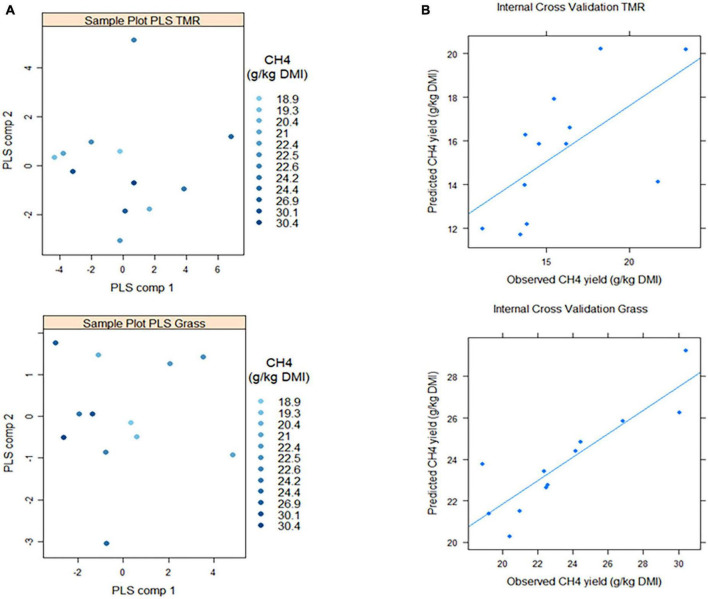
Results from linear projection of latent structures regression (PLS) models aiming to predict methane yield (g/kg DMI) based on 28 or 25 *clr*-transformed microbial genera in fresh cut grass (Grass) or high concentrate (TMR) fed animals. Full details of the PLS models can be found in Supplementary Table 2. **(A)** Sample plots (both PLS models were built with one single component, but two components were fitted only for visualization purposes). **(B)** After a threefold cross-validation procedure repeated 20 times, methane yield was predicted with 85 ± 8% (Grass) and 66.5 ± 9% prediction accuracy.

We fitted two multivariate PLS models (one with the GRASS animals and one with TMR-fed animals) with methane yield as the dependent variable and the 103 *clr*-transformed microbial genera as explanatory variables. In GRASS animals, a one-component PLS model built with the *clr*-transformed abundance of 28 microbial genera showed 85 ± 8% predictive ability for methane yield after 20 repetitions of 3-fold cross-validation (Supplementary Table 2; Figure 3). When the model was used to predict a randomized vector of methane yield in GRASS animals, the prediction ability decreased down to 36%. In animals fed a TMR diet, a single-component PLS model constructed with the *clr*-transformed abundance of 25 microbial genera showed 66.5 ± 9% predictive ability; and when methane yield was randomized, the prediction ability decreased down to 37%.

**FIGURE 4:**
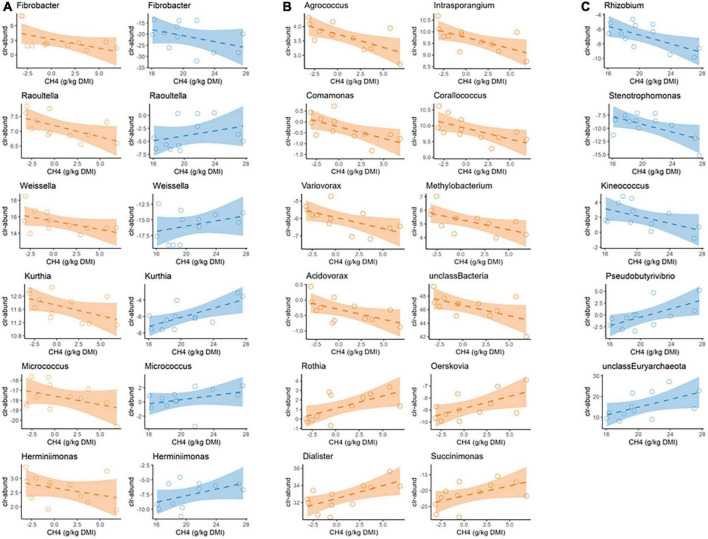
Visualization of linear associations between *clr*-transformed abundances of microbial genera identified with PLS analysis and methane yield in fresh cut grass (Grass, in blue) or high concentrate (TMR, in orange) fed animals. Full details of the analysis can be found in Supplementary Table 2. **(A)** Six microbial genera were selected in the PLS model in both diet groups, although only *Fibrobacter* was associated with methane yield in the same direction. **(B,C)** Microbial genera presenting a linear regression coefficient with a probability of being different from 0 ≤ 0.95 on methane yield only under TMR **(B)** or grass **(C)** diets.

We next examined whether there was a linear relationship between the *clr*-transformed abundance of these microbial genera and methane yield in a univariate approach (Supplementary Table 2). Of the 28 and 25 *clr*-transformed microbial genera selected in the PLS, only six genera were selected by the PLS algorithm as part of the model in both diets, indicating large differences in the microbiota compositions associated with methane yield in animals fed extremely different diets. Of the 6 *clr*-transformed microbial genera showing PLS predictive ability for methane yield under both diets, only the microbial genus *Fibrobacter* had a similar (negative) association with this trait (Figure 4A; Supplementary Table 2). Its *clr*- abundance showed a negative Pearson correlation with methane yield under both GRASS (−0.41 ± 0.26) and TMR (−0.53 ± 0.22) and a negative regression coefficient of −0.25 [−0.67, 0.15] (*P*_0_ = 0.89) and −0.75 [−1.63, 0.17] (*P*_0_ = 0.95), respectively. Although our PLS analysis did not select *Methanobrevibacter* as an optimal predictor of methane yield, we wanted to pay particular attention to it because its role in rumen methanogenesis is well known (Evans et al., 2019). The *clr*-abundance of *Methanobrevibacter* genus was positively associated with methane yield in both diets, but the s.e. of Pearson correlations were very large (0.25 ± 0.30 in GRASS and 0.41 ± 0.26 in TMR) and the probability of the linear regression coefficients of being positive were only moderate (in TMR it was 1.10 [−0.69, 2.88] *P*_0_ = 0.89 and in GRASS it was 0.65 [−1.12, 2.60] *P*_0_ = 0.77). Interestingly, the other five microbial genera expressing PLS predictive ability of methane yield in both diets were bacterial genera (*Weissella, Kurthia, Raoultella, Herminiimonas*, and *Micrococcus*) that suggests a changing association with methane yield depending on the diet. Their *clr*-transformed abundance was associated with a decrease in methane yield under TMR diet, with *Raoultella, Kurthia* and *Weissella* showing the strongest correlations with methane yield of −0.60 ± 0.20, −0.52 ± 0.23 and −0.52 ± 0.23, and regression coefficients of −4.74 [−9.58, −0.17] *P*_0_ = 0.98; −4.30 [−9.58, 1.18] *P*_0_ = 0.95 and −1.340 [−3.06, 0.22] *P*_0_ = 0.95, respectively. In contrast, their abundance was associated with an increase in methane yield within the GRASS diet, *Kurthia, and Herminiimonas* showing the strongest correlations with methane yield of 0.69 ± 0.17 and 0.49 ± 0.24 and regression coefficients of 1.71 [0.33, 3.09] *P*_0_ = 0.99 and 0.85 [−0.31, 2.01] *P*_0_ = 0.93, respectively (Figure 4A). These results suggest that the effect of abundance of a particular microorganism on methane yield may depend on diet-induced rumen environmental conditions.

The majority of the microbial genera showing predictive ability in PLS were different between diets (19/25 and 22/28 in TMR and GRASS, respectively). Of these, 12 and 5 showed a linear regression coefficient with methane yield that was different from 0 with a *P*_0_ ≥ 0.95 (Figures 4B, C). Under TMR conditions (Figure 4B; Supplementary Table 2), *clr*-transformed abundance of 12 bacterial genera, including five Proteobacteria (*Comamonas, Variovorax, Acidovorax, Corallococcus, and Methylobacterium*) and unclassified Bacteria, had a mitigation effect on methane yield (Pearson correlation of −0.58 ± 0.21 to −0.64 ± 0.19 and regression coefficient from −1.08 [−2.21, 0.10] to −5.75 [−11.4, −0.25], *P*_0_ ≥ 0.96), while abundance of the four bacterial genera (*Dialister, Rothia, Succinimonas*, and *Oerskovia*) was associated with an increase in emissions (Pearson correlation from 0.54 ± 0.22 to 0.67 ± 0.17 and regression coefficient from 0.46 [−0.13, 0.99] to 1.90 [−0.13, 3.65], *P*_0_ ≥ 0.95). Under GRASS conditions (Figure 4C; Supplementary Table 2), methane yield was positively correlated with the bacterial genus *Pseudobutyrivibrio* and the archaea Unclassified *Euryarchaeota* (Pearson correlation of 0.64 ± 0.19 and 0.57 ± 0.22 and regression coefficient of 0.89 [0.07, 1.74] and 0.34 [−0.01, 0.74] *P*_0_ ≥ 0.97), whereas the Proteobacterial genera *Stenotrophomonas* and *Rhizobium* and Actinobacterial genera *Kineococcus* were negatively associated (Pearson correlation from −0.54 ± 0.22 to −0.66 ± 0.18 and regression coefficient from −1.00 [−1.96, 0.01] to −1.41 [−2.66, −0.11], *P*_0_ ≥ 0.95).

## Discussion

Because of the large contribution of ruminants to total greenhouse gas emissions from livestock (Gerber et al., 2013), there is urgent interest in reducing enteric methane emissions and minimizing the environmental impact of beef and dairy farming. Beef cattle are raised worldwide under a variety of different conditions and feeding systems that need to be considered when developing methane mitigation strategies, especially if they are based on microbiome measurements, in which diet has a major impact. In this study, we provide insight into how microbial genera abundances associated with methane emissions differ between extreme diets, and therefore which pose the best proxies for predicting the trait under specific diet types. The influence of these extreme diets (high concentrate and fresh cut grass) on microbial biota have rarely been directly compared (McGovern et al., 2020), and never, as far as we are aware, with a low-quality grass.

As a target for mitigation, methane emissions can be expressed as daily production (g/day) or relative to inputs (e.g., methane yield, g/kg DMI) or outputs (e.g., methane intensity, g/kg of meat), among others (de Haas et al., 2017). The obvious issue with targeting methane production is that it is correlated with feed intake. Our study showed that methane yield was more closely related to microbiota parameters (A:B) than methane production (g/day), especially on the poor quality grass diet, and therefore it might be more appropriate to consider it as a methane measurement when searching for microbiome proxies targeting methane mitigation.

As expected, diet had a strong effect on methane yield (Lovett et al., 2003; Aguerre et al., 2011; Troy et al., 2015; Olijhoek et al., 2018). Animals fed low quality fresh cut grass had 10.6 g of methane/kg of dry matter intake (73%) higher methane yield than those fed a high concentrate TMR. This is well explained in the literature by the passage rate of grain solids being faster than those of forage, and concentrate diets being richer in starch and proteins. Higher passage rate, together with fermentation of starch, are associated with fermentation pathways that lead to more propionate and less hydrogen available for archaea to reduce carbon dioxide to methane, in comparison to slower passage rates and fermentation of fiber in forage diets (Wolin, 1979; Janssen, 2010). Diet composition is also a critical factor in microbial ecology (Gruninger et al., 2014), as it determines the rumen conditions that favor the adaptation and growth of specific microbial species. Our extreme diets altered the ratio between the abundance of archaea and bacteria, with the poor quality fresh cut grass having a detrimental impact on the bacterial community.

Diet had a strong influence on the composition of the microbiota, even when analyzed at lower taxonomic levels, with 28 genera perfectly discriminating between the two extreme diets. However, the observed changes in methane yield and microbiota composition caused by the diet were not necessarily related. For example, we found that animals fed a poor quality fresh cut grass had significantly increased relative abundance of *Bacillus*, *Lactococcus, Pseudomonas, Microbacterium*, and unclassified *Actinobacteri*a and a significant decrease in *Mitsuokella, Planococcus*, *Lactobacillus, Desulfovibrio*, and *Aerococcus* compared with TMR-fed animals. However, none of these abundances were selected in a PLS model to predict methane yield in each diet group. This can be explained by the fact that diet alters rumen conditions, which affects the microbial genera associated with methane emissions, but also other non-related microbial genera. It should be noted that these results may reflect an actual lack of methane-microbe associations, or a minor effect not captured in this study due to lack of power (loss of data points due to mechanical chamber failure).

The lack of a strong correlation of methane yield and *Methanobrevibacter* abundance could be due to the fact that different *Methanobrevibacter* strains classified within *Methanobrevibacter* genera are functionally versatile and therefore have different effects on methane yield, as observed in Kittelmann et al. (2013) and Martínez-Álvaro et al. (2022). In other cases, diet significantly altered the abundance of certain microbial genera that were associated with methane yield (selected in PLS) and therefore could help explain the 73% difference between diets in methane yield. For example, the abundance of Unclassified Bacteria was relevantly increased in the high concentrate diet and had a significant mitigation effect on methane yield on this diet group. On the other hand, *Bifidobacterium* was increased in the high concentrate diet but had a positive effect on methane emissions under a poor quality fresh cut grass diet, although the regression coefficient was different from zero with only a moderate probability. *Bifidobacterium* species produce lactic acid and acetic acid, fermentation products usually associated with increased hydrogen production, which potentially increases the synthesis of methane (Moss et al., 2000; Danielsson et al., 2017). Another example is the abundance of the dominant cellulolytic genus *Fibrobacter*, which was increased in animals fed the low-quality grass diet and showed predictive ability and a negative correlation with methane yield in both diet groups, with stronger effect in the high concentrate diet. In their study, Kittelmann et al. (2013) inferred a co-occurrence of bacteria from the *Fibrobacteaceae* family, including the major cellulolytic bacterium *Fibrobacter succinogenes*, which produces only formate and no H_2_ (Rychlik and May, 2000), and hydrogenotrophic- methanogenesis was reduced (Smith and Hungate, 1958; Leahy et al., 2013). In contrast, bacteria from the *Ruminococcaceae* family, some of which produce large amounts of H_2_ (e.g., *Ruminococcus* sp.), co-occurred with *M. gottschalkii*, which is capable of producing methane from H_2_ and CO_2_ but not from formate (Miller and Lin, 2002). Later, the same authors associated greater abundance of *Fibrobacter* sp. with a low methane emitting ruminotype and greater abundance of *sp.* from *Ruminococcus* genus and other *Ruminococcaceae* with a high methane emitting ruminotype (Kittelmann et al., 2014). These studies may explain our negative association between *Fibrobacter* and methane yield observed in both diets; although *Ruminococcus* genus abundance was not associated with methane yield in any case (data not shown).

Except for *Fibrobacter*, the few microbial genera that were predictive of methane yield under both diets had different effects on methane yield. In addition, most microbial genera that were predictive of methane yield differed between diets, suggesting that the main microbial drivers of methane yield depend on the products available for substrates microbial fermentation. On the high concentrate diet, several genera from the *Proteobacteria* phylum (*Acidovorax, Comamonas, Corallococcus, Variovorax*, and *Methylobacterium*) were negatively associated with methane yield along with other unclassified Bacteria. In the low-quality fresh cut grass group, some Proteobacteria were also negatively associated with methane yield, but they belonged to other genera (*Stenotrophomonas* and *Rhizobium*). The abundance of some of these genera from the Proteobacteria phylum has been negatively associated with methane in the literature (Wallace et al., 2015; Danielsson et al., 2017; Auffret et al., 2018; Martínez-Álvaro et al., 2020). The explanation might be based on the fact that these *Proteobacteria* genera are methanotrophic in the rumen (e.g., *Methylobacterium*); or capable of fixing nitrogen (e.g., *Rhizobium*), at least in the host plant (Munoz Aguilar et al., 1988). In the rumen, N_2_ reduction to ammonia may act as an alternative H_2_ consuming sink competing with ruminal methanogenesis (Bulen and LeComte, 1966). On a low-quality fresh cut grass diet, unclassified *Euryarchaeota*, *Pseudobutyrivibrio* and *Kurthia* had a strong effect increasing methane emissions, which we did not observe in the high concentrate diet. Instead, *Succinimonas*, *Oerskovia, Rothia* and *Dialister* had the positive and strongest effect increasing methane yield. Methanogenic archaea belong to the *Euryarchaeota* phylum, while *Pseudobutyrivibrio* plays an important role in the release of formate (Tapio et al., 2017) and other by-products such as butyrate, known to be associated with an increase in methane emissions (Kamke et al., 2016). Only in a few cases did we observed a changing effect of the abundance of a particular microbe under different diets (e.g., *Kurthia* or *Weissella*, see Figure 4A); but only for *Kurthia* the regression coefficients were different from 0 in both diets and allows to confirm a diet-microbiota cross-over interaction.

In this study, we found that different microbes drive emissions when hosts are fed with diets extreme in their forage to concentrate ratio; therefore, different mitigation strategies targeting specific microbial mechanisms must be adequate to each feeding system. To date, targeted sequencing of specific regions of the 16S gene is the most cost-effective method, although it may not provide high taxonomic resolution compared to metagenomic sequencing. Nevertheless, in our study, we achieved high accuracy in predicting methane yield for both the poor-quality grass and high concentrate TMR dietary scenarios, with a PLS algorithm using almost all different microbial abundances. Our results are most relevant to the development of strategies to reduce methane emissions based on changes in the microbiome, as different diets determine which microbial taxa have the greatest impact on methane yield, and diet-specific strategies should be considered. Our study is a small-scale study with the main goal of identifying the difference in microbiota compositions between two extreme diets and its use for prediction of methane emissions. This study draws attention to the need for different mitigation strategies adapted to different diet types, but further larger scale studies are required to understand diet-microbiome interactions that influence methane emissions. Also, microbiome based methane predictions need to ben locally validated the ensure factors such as local dietary factors are not skewing the results.

## Conclusion

The divergent impacts on the rumen microbial composition of extreme diets, in this case high concentrate and low-quality grass, have rarely been directly compared. These differences in microbial composition can be used to predict methane yield of individual animals. Using two groups of beef cattle fed with two extreme diets on their forage to concentrate ratio, we found a reduced set of microbial genera with a high predictive accuracy for methane yield of 85 and 66% in the forage or concentrate-based diets. Among the microbial genera that predicted methane yield, there was little overlap between diets, and most microbial genera were diet specific. This finding is critical for the development of mitigation strategies based on the microbiome, where, according to this study, diet-specific strategies should be considered at least at phenotypic level.

## Data availability statement

The datasets presented in this study can be found in online repositories. The names of the repository/repositories and accession number(s) can be found below: https://www.ebi.ac.uk/ena/browser/home, PRJEB57703.

## Ethics statement

This animal trials described below were approved by the Animal Experiment Committee of SRUC and were conducted in accordance with the requirements of the UK Animals (Scientific Procedures) Act 1986.

## Author contributions

GM: conceptualization, methodology, investigation, and writing—original draft. MA: conceptualization, methodology, review, and editing. RR: conceptualization, supervision, review, and editing. HN: methodology and investigation. MM-Á: software, formal analysis, and writing—original draft. All authors reviewed and approved the manuscript.

## Footnotes

1
https://github.com/VLabUPV/runRabbit
