# Supplementary material for: Different microbial genera drive methane emissions in beef cattle fed with two extreme diets
Source: Front Microbiol. 2023 Apr 13;14:1102400. doi: 10.3389/fmicb.2023.1102400 (PMC10133469; doi:10.3389/fmicb.2023.1102400)
Supplement: Supplementary file 1 [file Data_Sheet_1.PDF]

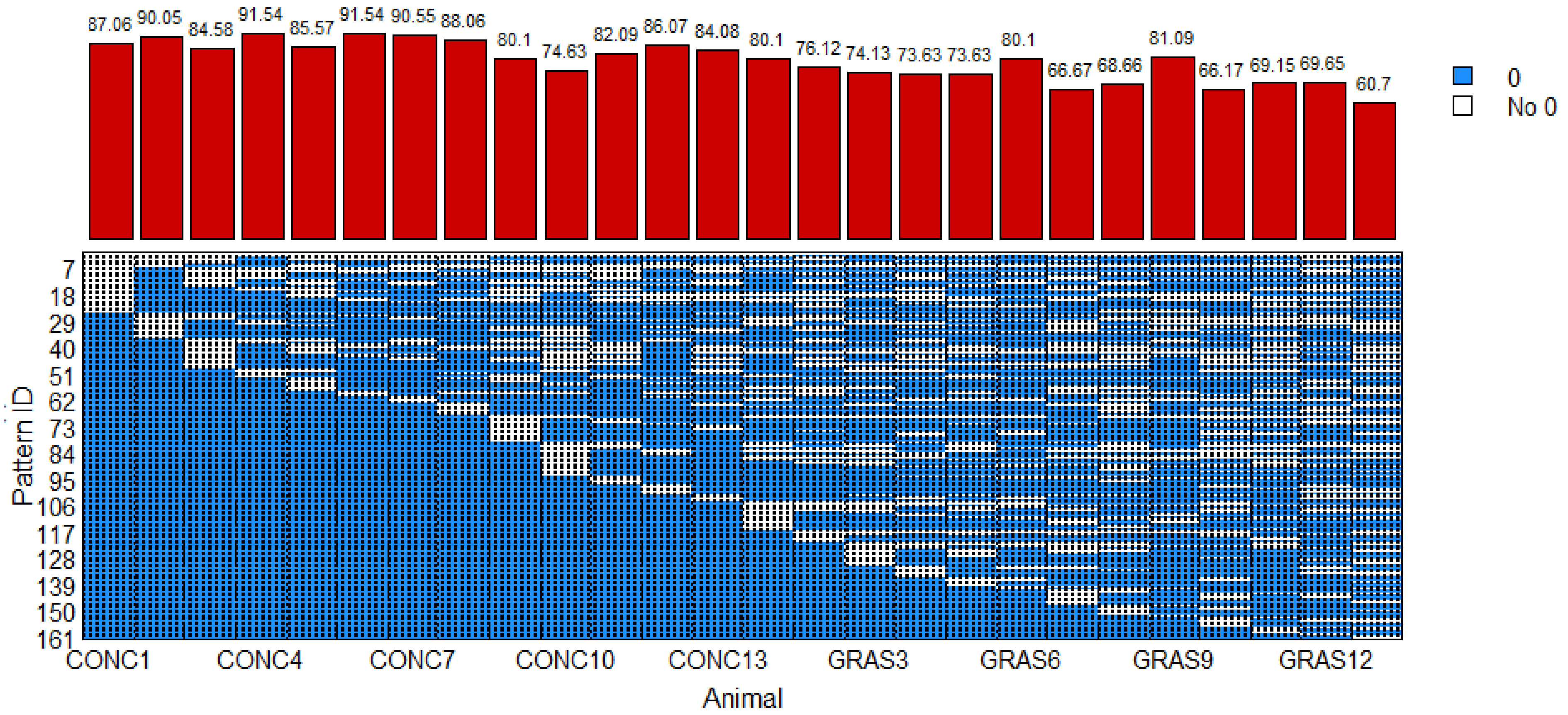

Supplementary Figure 1: Amount of 0 counts identified on each of the 26 samples when the rumen taxonomical composition is described at genera level.

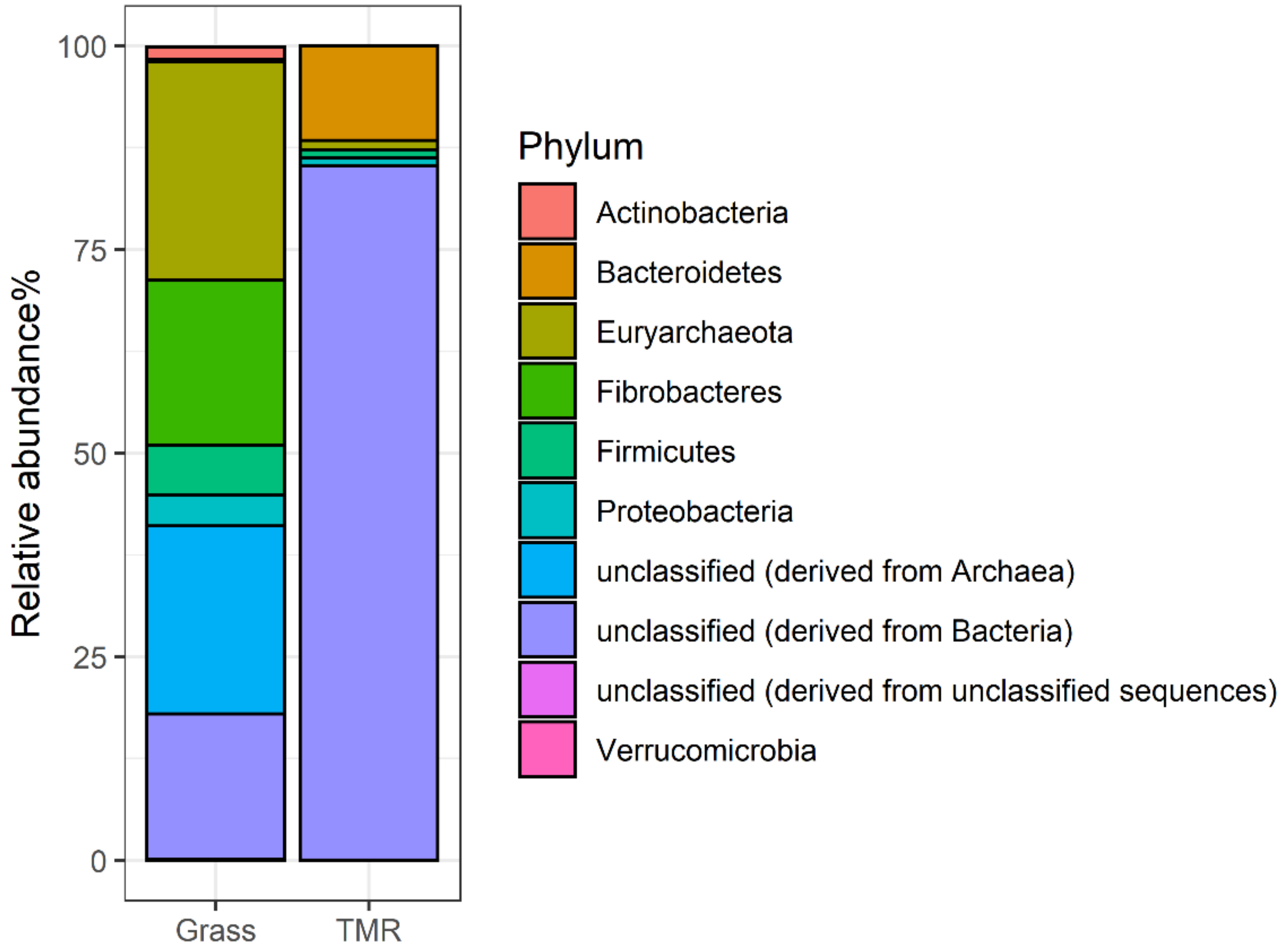

Supplementary Figure 2: Rumen microbiome taxonomic composition at phylum of level of the groups of animals fed with total-mixed based ration (TMR) or Grass.
